# Efficient Intracellular Delivery of Cell-Impermeable Cargo Molecules by Peptides Containing Tryptophan and Histidine

**DOI:** 10.3390/molecules23071536

**Published:** 2018-06-26

**Authors:** Amir Nasrolahi Shirazi, Saghar Mozaffari, Rinzhin Tshering Sherpa, Rakesh Tiwari, Keykavous Parang

**Affiliations:** Center for Targeted Drug Delivery, Department of Biomedical and Pharmaceutical Sciences, Chapman University School of Pharmacy, Harry and Diane Rinker Health Science Campus, Irvine, CA 92618, USA; nasro100@mail.chapman.edu (A.N.S.); mozaf100@mail.chapman.edu (S.M.); sherp101@mail.chapman.edu (R.T.S.)

**Keywords:** histidine, Peptide-based Drug Delivery Systems, phosphopeptides, tryptophan

## Abstract

We have previously evaluated and reported numerous classes of linear and cyclic peptides containing hydrophobic and hydrophilic segments for intracellular delivery of multiple molecular cargos. Herein, a combination of histidine and tryptophan amino acids were designed and evaluated for their efficiency in intracellular delivery of cell-impermeable phosphopeptides and the anti-HIV drug, emtricitabine. Two new decapeptides, with linear and cyclic natures, both containing alternate tryptophan and histidine residues, were synthesized using Fmoc/tBu solid-phase chemistry. The peptides were characterized and purified by using matrix-assisted laser desorption/ionization (MALDI) spectroscopy and high-performance liquid chromatography (HPLC), respectively. These peptides did not show significant toxicity up to 100 µM in ovarian cancer (SK-OV-3) and leukemia cancer (CCRF-CEM) cells. Furthermore, the cellular uptake of a fluorescence (F’)-labeled cell-impermeable phosphopeptide (F’-GpYEEI) was enhanced in the presence of linear (WH)_5_ and cyclic [WH]_5_ by 2- and 8-fold, respectively, compared to the uptake of the phosphopeptide alone. The cellular uptake was not significantly changed in the presence of endocytosis inhibitors. Furthermore, the intracellular uptake of the fluorescently-labeled anti-HIV drug, emtricitabine (F’-FTC), by linear (WH)_5_ and cyclic [WH]_5_ in SK-OV-3 cancer cell lines was found to be enhanced by 3.5- and 9-fold, respectively, compared to that of the drug alone. Fluorescent uptake experiments confirmed the localization of F’-GpYEEI-loaded cyclic [WH]_5_ intracellularly in the SK-OV-3 cancer cell line after 3 h of incubation. Thus, these data demonstrated that [WH]_5_ containing tryptophan and histidine enhanced the cellular uptake of F’-GpYEEI and emtricitabine.

## 1. Introduction

Peptide-based Drug Delivery Systems (PDDS) have been introduced as one of the prominent non-viral delivery tools of various types of cargo molecules [1]. PDDS are capable of loading molecules with relatively low molecular weight, such as doxorubicin, lamivudine, and dasatinib [2,3], and molecules with relatively high molecular weight, such as siRNA, phosphopeptides, and DNA [4,5]. The majority of PDDS possess a fine balance of physicochemical properties based on their assigned tasks [6]. They can be categorized as amphipathic, hydrophobic, and cationic peptides [7]. Furthermore, numerous PDDS, including Tat peptide, polyarginine, and penetratin, have been developed for the delivery of drugs [8]. 

Herein, we have used a phosphopeptide to model a cell-impermeable cargo molecule. Phosphopeptides are employed as probes in signaling transduction studies due to their ability to mimic cellular phosphoproteins [9]. Phosphopeptides have been used to understand protein-protein and protein-ligand interactions [10] due to their specificity as substrates to different binding domains in protein kinases, for example, the Src homology 2 (SH_2_) domain in Src tyrosine kinase [11]. The application of phosphopeptides in such experiments has been a challenging task due to their restricted cellular uptake. Their limited uptake is due to the presence of negatively charged phosphate groups in their chemical structures, causing electrostatic repulsion with the negatively-charged phosphate groups in the phospholipid bilayer.

PDDS can take advantage of multiple mechanisms to transport molecular cargos into cells. They can undergo either endocytosis-mediated pathway or direct translocation. Moreover, PDDS can be used for targeted delivery of small to medium sized drugs, such as anti-HIV and anti-cancer drugs. We have previously reported enhanced efficiency of tryptophan and arginine containing [WR]_5_ as PDDS containing positively-charged and hydrophobic amino acids in transporting anti-HIV and anticancer drugs. It was discovered that the presence of both arginine and tryptophan amino acids were critical to the function of the carrier system. Further investigations showed that, although the number of amino acids was important to maximize the transporting ability of the peptide, an optimized balance between electrostatic and hydrophobic forces are crucial for their high yield of intracellular uptake into cells [2,6,12,13,14,15,16,17].

As described above, the number of involved amino acids and their orientation in the structure of PDDS could impact their delivery efficiency significantly. After evaluation of numerous cyclic and linear peptides for their efficiency to deliver molecules and/or drugs intracellularly, it was discovered that an alternative sequence of amino acids offers prominent advantages in terms of their transporting potency [2]. 

The presence of hydrophobic segments in the structure of PDDS induces high affinity to the hydrophobic portion of the cell membrane, leading to translocation across the lipid membrane through energy-independent pathways [18]. In addition to the required hydrophobicity in the structure of PDDS, positively charged amino acids were found to be engaged in generating and maintaining electrostatic and hydrogen bonding interactions. Therefore, the presence of arginine residues was discovered to be critical to enhance the cell-penetrating efficiency of the peptide. 

As novel PDDS are being developed through new investigations, there have been more efforts to make them multifunctional. For instance, several PDDS have been reported to deliver molecular cargos, such as drugs [19], nanoparticles [20], proteins [21], DNAs [22], and siRNAs [23], to specific site of actions. Among the reported peptides, gH625 (HGLASTLTRWAHYNALIRAF) was found to be an efficient transporting tool to deliver cargo molecules into the cytoplasm. The peptide, gH625, was used to carry quantum dots into the cytoplasm efficiently through the endocytosis pathway [24]. Furthermore, numerous chimeric amphipathic peptides have been introduced for nuclear delivery into cells [25].

Histidine-rich PDDS have been found to be rather interesting due to the ability of histidine to respond to acidic medium. For instance, a histidine-rich PDDS, namely “TH”, is a sequence of amino acids, which is rich in the histidine, AGYLLGHINLHHLAHL(Aib)HHIL-NH_2_ [26]. It was found that the presence of an alkylated histidine moiety in the structure of TH could enhance the protonation process in weakly acidic media. This would theoretically empower the PDDS to be used for targeting cancer cells, with a higher degree of toxicity compared to normal cells. 

In addition to histidine, tryptophan has been used in the structure of peptides due to its unparalleled biophysical and chemical properties. The ability of tryptophan to encapsulate, entrap, and facilitate hydrophobic molecular cargos could be a significant advantage [6]. Furthermore, tryptophan promotes hydrophobic interactions with the cell membrane, leading to enhanced penetration of the peptide into the lipid bilayer system. It was found that, at least three tryptophan amino acids are required to be present in the structure of the peptide to obtain an effective tryptophan-based drug delivery system. However, by adding an extra tryptophan into the peptide’s structure, the peptide’s cellular uptake could decrease significantly, showing that solubility limitations can be a barrier to the functionality of peptides [27]. 

To overcome the solubility barrier for peptides containing only tryptophan, we have previously used positively-charged arginine with tryptophan in an alternative sequence model, leading to the improvement of the aqueous solubility in the physiological environment [2]. In continuation of our efforts to discover novel PDDS for efficient transportation of molecular cargos, herein, we explored peptides containing tryptophan and histidine residues. The hypothesis was that the presence of hydrophobic segment composed of tryptophan could create a hydrophobic pocket and enhance the transportation of molecular cargos through the lipid bilayer, leading to a higher uptake. Thus, we report a new class of decapeptides containing an alternative sequence of tryptophan and histidine amino acids as efficient intracellular carriers. To the best of our knowledge, this is the first report of using a combination of alternate histidine and tryptophan amino acids in the structure of a short peptide for drug delivery applications.

## 2. Results and Discussion

### 2.1. Design and Synthesis of Linear and Cyclic Peptides

**Scheme 1:**
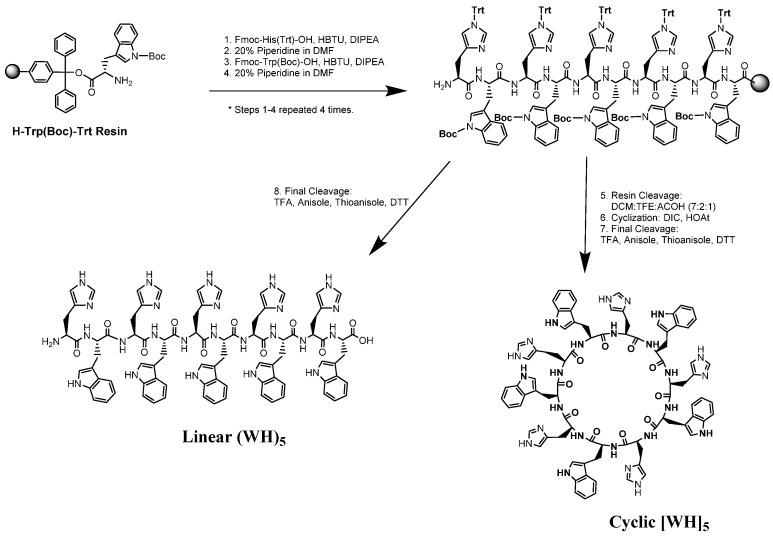
Synthesis of linear (WH)_5_ and cyclic [WH]_5._

Two cyclic and linear peptides containing five alternative l-histidine and l-tryptophan amino acids (Scheme 1), [WH]_5_ and (WH)_5_, were synthesized with solid-phase chemistry using 9-fluorenylmethyloxycarbonyl (Fmoc)/t-Bu, according to the previously reported procedure [13]. 2-Chlorotrityl resin preloaded with tryptophan was used as the solid support for peptide synthesis. All peptides were purified by reverse-phase high-performance liquid chromatography (RP-HPLC) and analyzed by matrix-assisted laser desorption/ionization time of flight (MALDI-TOF/TOF) mass spectroscopy (see Supplementary Materials). 

Scheme 1 depicts the synthetic procedure including several steps of reactions. Assembly of side-chain protected histidine and tryptophan on the solid support followed by complete cleavage afforded the linear peptide. Furthermore, partial cleavage from the resin was used to generate the side-chain protected peptide for cyclization and side chain deprotection, according to our previously reported methods [28]. 

### 2.2. Cytotoxicity of Peptide-Based Drug Delivery Systems

**Figure 1:**
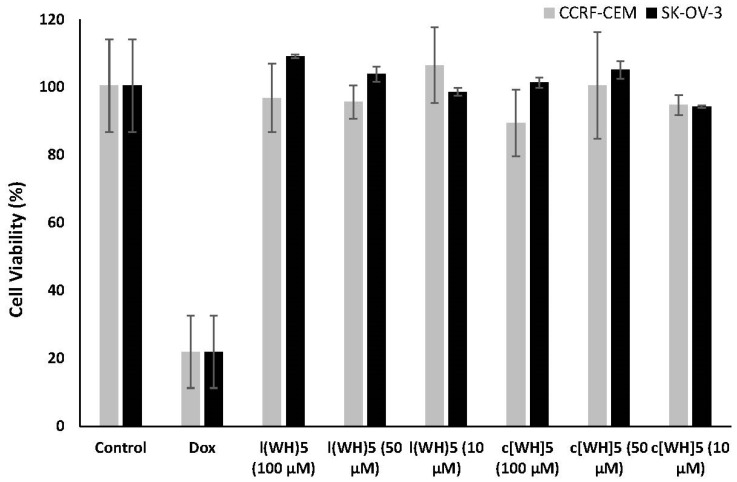
Cytotoxicity for linear (WH)_5_ and cyclic [WH]_5_ in human ovarian (SK-OV-3) and leukemia (CCRF-CEM) cells after 3 h of incubation. Doxorubicin (Dox: 5 µM) was used as a control.

To determine concentration of peptide to be used in in vitro assays, the cytotoxicity of peptides was evaluated in two cell lines, human ovarian (SK-OV-3) and leukemia (CCRF-CEM) cancer cells. Different concentrations of peptides (10, 50, and 100 µM) were incubated with cells for 3 h (Figure 1). Both linear (WH)_5_ and cyclic [WH]_5_ did not show significant toxicity at the concentration of up to 100 µM in CCRF-CEM and SK-OV-3 cells. As shown in Figure 1, there was no significant difference between linear and cyclic peptides. Our results suggested that these peptides do not exhibit any significant toxicity up to 100 µM. Thus, a concentration of 50 µM was selected as a safe non-toxic one to be used for further biological assays.

### 2.3. Cellular Uptake of Fluorescence-Labeled Phosphopeptide (F’-GpYEEI) by Flow Cytometry

To assess the potential of cyclic [WH]_5_ and linear (WH)_5_ as PDDS, a model experiment was designed using a cell-impermeable phosphopeptide. As explained previously, phosphopeptides are important biomolecules that suffer from limited cellular uptake due to the presence of the negatively charged phosphate group in the structure. Thus, a phosphopeptide, GpYEEI, was selected as the molecular cargo for this experiment. To be able to monitor the uptake of the phosphopeptide, a fluorescent label was attached to the phosphopeptide to generate F’-GpYEEI (where F’ represents carboxyfluorescein and pY represents the phosphotyrosine amino acid) [17]. SK-OV-3 cells were incubated with F’-GpYEEI (5 μM) in the presence and absence of linear (WH)_5_ and cyclic [WH]_5_ (50 μM) for 3 h at 37 °C. After 3 h of incubation, trypsin was added to remove the extracellular-bounded F’-GpYEEI, leading to a better evaluation of the intracellular uptake.

**Figure 2:**
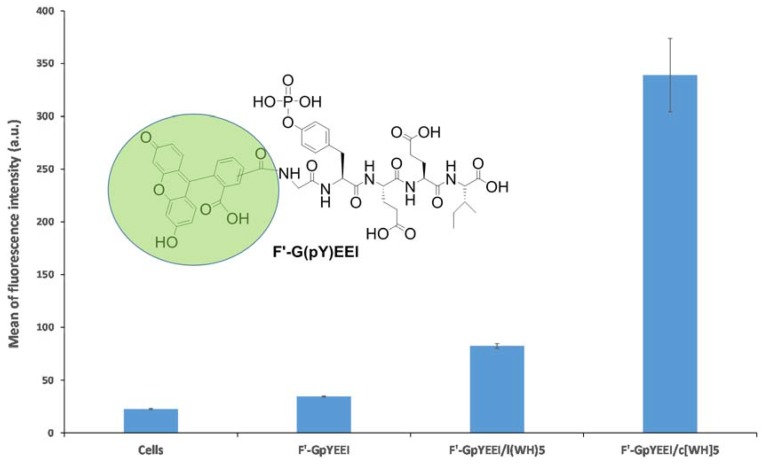
Intracellular uptake of F’-GpYEEI by linear (WH)_5_ and cyclic [WH]_5_ in SK-OV-3 cancer cell lines.

A fluorescence-activated cell sorter (FACS) was used to quantify the intracellular uptake of F’-GpYEEI. Here, the results showed that a higher fluorescence signal of F’-GpYEEI existed in cells in the presence of peptides. For instance, the cellular uptake of F’-GpYEEI was enhanced by c[WH]_5_ and l(WH)_5_, 9- and 2.3-fold, respectively, as compared to that of the phosphopeptide alone (Figure 2). Cells alone were also used as a negative control to evaluate the auto-fluorescence inside cells. These peptides were able to improve the intracellular delivery of the phosphopeptide, possibly due to the formation of a complex with F’-GpYEEI, as we described previously [2,17]. The data were consistent with the previous work [2] showing that the cyclic peptide-based drug delivery system functions at a higher efficiency compared to its linear counterpart.

In addition to the phosphopeptide (F’-GpYEEI), a fluorescence-labeled anti-HIV drug (F’-FTC) was also tested as molecular cargo to evaluate the efficiency of the peptides in delivering emtricitabine (FTC), an anti-HIV nucleoside reverse transcriptase inhibitor. FTC blocks HIV-1 and hepatitis B virus replication [29]. Although FTC proved to be a potent antiviral agent, it has limited cellular uptake. Here, a carboxyfluorescein derivative of FTC (F’-FTC) was synthesized as previously reported [30].

**Figure 3:**
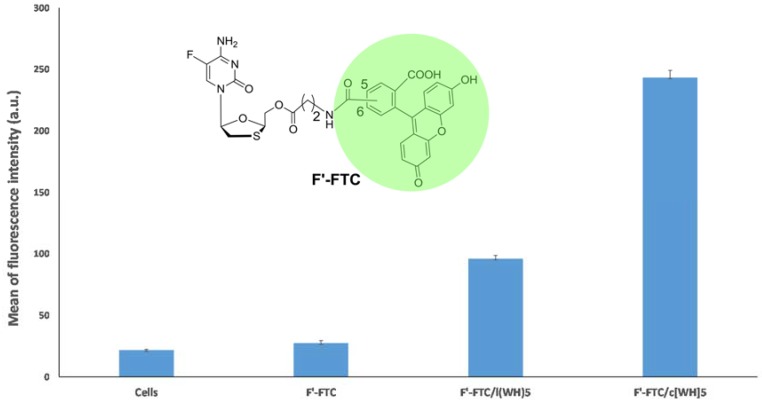
Intracellular uptake of F’-FTC by linear (WH)_5_ and cyclic [WH]_5_ in SK-OV-3 cancer cell lines.

A similar experiment was carried out for F’-FTC. SK-OV-3 cells were incubated with F’-FTC (5 μM) alone and in combination with the peptides (linear and cyclic (WH)_5_) (50 μM) in different wells. Cells incubated with F’-FTC alone was used as a control. The fluorescence of F’-FTC was showed to be significantly enhanced in the presence of c[WH]_5_ by ~9 folds compared to that of F’-FTC alone (Figure 3). Moreover, linear (WH)_5_ increased the cellular uptake of F’-FTC by ~3.5 folds compared to that of F’-FTC alone. The data showed that both linear and cyclic peptides promoted the cellular uptake of F’-FTC. However, the cyclic c[WH]_5_ enhanced the cellular uptake of the drug at a higher level compared to the linear (WH)_5_ peptide. It is also important to determine the biological activity of the cargo molecules in the presence of the peptide as the delivery tool. We have formerly shown the biological activity of doxorubicin in the presence of peptides containing tryptophan and arginine resides [28]. However, the objective of the present work was to evaluate peptides containing tryptophan and histidine as delivery tools. The evaluation of the biological activity of the cargo drugs is beyond the scope of the current work.

### 2.4. Cellular Uptake of F’-GpYEEI in the Presence of Inhibitors

**Figure 4:**
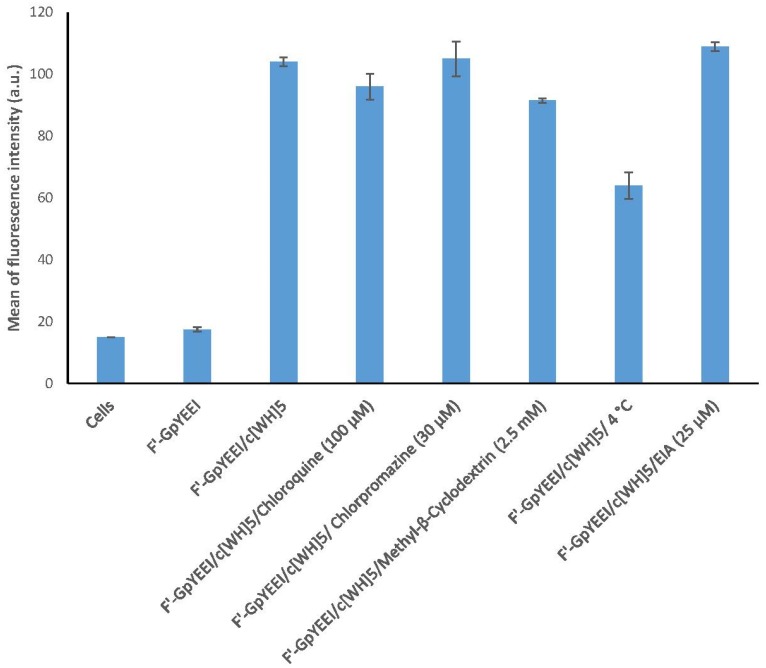
Intracellular uptake of F’-GpYEEI by cyclic [WH]_5_ in CCRF-CEM cancer cell lines.

Molecular cargos utilize different mechanism pathways to enter cells. These mechanisms could include, but are not limited to, receptor-mediated endocytosis (RME), phagocytosis, and micropinocytosis pathways. RME pathways could involve Clathrin or Caveolae elements for transportation. As shown in Figure 4, the cellular uptake of F’-GpYEEI in the presence of [WH]_5_ was not found to be significantly impacted in the presence of chloroquine, chlorpromazine, methyl-β-cyclodextrin, and EIA after 3 h of incubation at 37 °C in CCRF-CEM cells, suggesting that the uptake is not mainly mediated by clathrin or caveolae pathways in endocytosis and phagocytosis [2,17]. In addition, when EIA was used, the cellular uptake of F’-GpYEEI did not alter noticeably, revealing that macropinocytosis pathways are not primarily reasonable for the cellular uptake. However, the cellular uptake of F’-GpYEEI was reduced by 40% when cells were incubated at 4 °C compared to that at 37 °C (Figure 4). These results showed that, in addition to direct penetration, there could be energy-dependent pathways involved in improving the uptake.

### 2.5. Cellular Uptake of F’-GpYEEI by Microscopy

**Figure 5:**
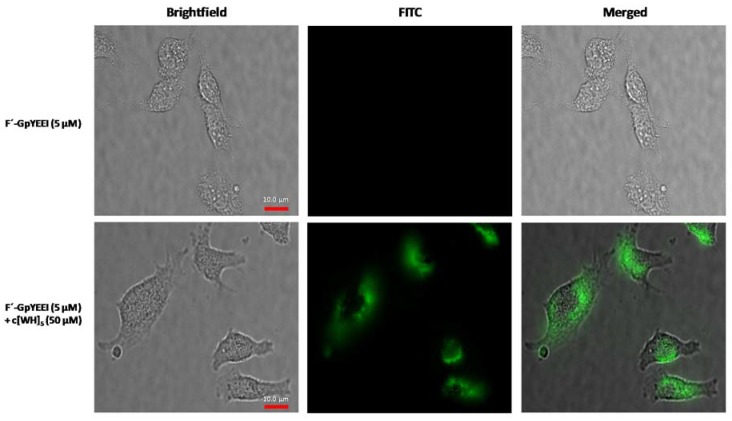
Microscope images of F’-GpYEEI uptake by SK-OV-3 cells in the presence of cyclic [WH]_5_ after 3 h incubation. No green fluorescence was observed in the presence of F’-GpYEEI alone. FITC: Fluorescein isothiocyanate.

Fluorescence microscopy was used to confirm the intracellular uptake of F’-GpYEEI in the presence of cyclic [WH]_5_. The fluorescence intensity of F’-GpYEEI (5 µM) was visualized in a physical mixture with cyclic [WH]_5_ (50 µM) compared to that of F’-GpYEEI alone in SK-OV-3 cells. Microscopy results showed the intracellular localization of F’-GpYEEI-loaded cyclic [WH]_5_ after 3 h of incubation. F’-GpYEEI alone was used as a control in this assay. Live cell imaging was carried using glass-bottom plates. This data verified that the peptide enhances the cellular uptake of F’-GpYEEI, suggesting a higher cellular permeability of the molecular cargo in the presence of [WH]_5_ (Figure 5). F’-GpYEEI was observed to be localized in the cytosol.

## 3. Materials and Methods

### 3.1. General Information

All amino acids and reagents were prepared from Chem-Impex International, Inc. and Sigma-Aldrich Chemical Co. (Milwaukee, WI, USA). High-resolution MALDI ABX SCIEX TOF/TOF 5800 was used for the characterization of the peptides. Assembly of amino acids was carried out in Bio-Rad polypropylene columns under nitrogen atmosphere [20,21,22,23]. The *N*-(9-fluorenyl)-methoxycarbonyl (Fmoc)-based chemistry was used for the peptide synthesis. Fmoc-l-amino acid building blocks were employed to synthesize peptides. Linear peptide (WH)_5_ was synthesized through solid-phase synthesis. The NH_2_-Trp(Boc)-2-chlorotrityl resin was employed as the solid phase support. The 2-(1H−Benzotriazole-1-yl)-1,1,3,3-tetramethyluronium hexafluoro phosphate (HBTU) and *N*,*N*-diisopropylethylamine (DIPEA) in *N*,*N*-dimethylformamide (DMF) were used for coupling and activating the amino acids. The Fmoc deprotection process was carried out in the presence of piperidine in DMF (20%). Trifluoroethanol (TFE)/acetic acid/dichloromethane (DCM) (2:2:6, *v*/*v*/*v*, 15 mL) was washed for 2 h to obtain side-chain protected peptides. A mixture of 1-hydroxy-7-azabenzotriazole (HOAt) and *N*,*N*′-diisopropylcarbodiimide (DIC) in dry DMF and dry DCM was used to carry out the cyclization of the peptides for 24 h. The solvents (DMF and DCM) were evaporated after the completion of cyclization. The deprotection of the side chain was performed with trifluoroacetic acid (TFA)/thioanisole/anisole/1,2-ethanedithiol (EDT) (90:5:2:3 *v*/*v*/*v*/*v*) for 5 h. The peptides were precipitated by adding cold diethyl ether (Et_2_O). Final peptides were purified on a Waters C18 reverse phase column 10 μm ODS (2.1 cm × 25 cm) with a Hitachi HPLC system using a gradient system of 0–100% water (H_2_O) and acetonitrile (CH_3_CN) at a pH of 7.0 over 60 min. The synthesis of linear (WH)_5_ and cyclic [WH]_5_ is described below.

#### 3.1.1. Synthesis of Linear (WH)_5_ Peptide

Linear decapeptide containing alternative histidine and tryptophan residues (WHWHWHWHWH) was synthesized by Fmoc/tBu solid-phase peptide synthesis. H-Trp(Boc)-2-chlorotrityl resin (0.4 mmol, 1081 mg, 0.37 mmol/g) was swelled in DMF for 30 min under nitrogen. Fmoc-His(Trt)-OH (744 mg, 1.2 mmol) and Fmoc-Trp(Boc)-OH (632 mg, 1.2 mmol) were coupled alternatively to NH_2_-Trp(Boc)-2-chlorotrityl resin in the presence of HBTU (341 mg, 0.9 mmol) and DIPEA (315 µL, 1.8 mmol) in DMF. Fmoc groups were deprotected using 20% piperidine/DMF under nitrogen for two times (20 min × 2). Coupling and deprotection cycles were repeated to synthesize the sequence of the linear protected peptide. The side-chain deprotection and cleavage from the resin were then carried out by a cleavage cocktail reagent, “R” (TFA/thioanisole/anisole/1,2-ethanedithiol (EDT), 92:3:2.5:2.5, *v:v:v:v*, 15 mL), for 5 h. The crude peptide was precipitated by the addition of cold diethyl ether (75 mL, Et_2_O) and purified by reversed-phase Hitachi HPLC (L-2455) on a water X Bridge TM BEH130 Prep C18 OBD 10 μm ODS reversed-phase column (2.1 cm × 25 cm) using a gradient system. The crude peptide was purified at a flow rate of 10.0 mL/min using a gradient of 0–100% acetonitrile (0.1% TFA) and water (0.1% TFA) over 60 min on the RP-HPLC and was then lyophilized to obtain the linear peptide. L(WH)_5_: MALDI-TOF (*m*/*z*) for C_85_H_87_N_25_O_11_: calcd. 1633.7017; found 1634.8291 [M + H]^+^.

#### 3.1.2. Synthesis of Cyclic [WH]_5_ Peptide

The linear peptide was assembled on H-Trp(Boc)-2-chlorotrityl resin (0.4 mmol, 1081 mg, 0.37 mmol/g). After swelling in DMF for 30 min under nitrogen, Fmoc-His(Trt)-OH (744 mg, 1.2 mmol) and Fmoc-Trp(Boc)-OH (632 mg, 1.2 mmol) were coupled alternatively as described above to synthesize the linear peptide assembled on the resin. The side chain protected peptide was cleaved from the resin by shaking the resin with a mixture of TFE/acetic acid/ DCM (2:2:6, *v*/*v*/*v*, 50 mL) for 2 h. The resin was filtered off, and the solution was evaporated to dryness under reduced pressure to yield the side-chain protected linear peptide. Then, cyclization of the linear peptide was carried out in the presence of DIC (140 μL, 0.9 mmol) and HOAt (122.5 mg, 0.9 mmol) in dry DMF/DCM (200 mL, 3:1 *v*/*v*) under nitrogen and stirring for 12 h. After cyclization, the solvent was evaporated, and the side chain deprotection was performed by addition of TFA/thioanisole/anisole/EDT (92:3:2.5:2.5, *v:v:v:v*, 15 mL) and shaking on a shaker for 5 h. The crude peptide was precipitated by the addition of cold diethyl ether (75 mL, Et_2_O) and purified by reversed-phase Hitachi HPLC (L-2455) on a Waters XBridgeTM BEH130 Prep C18 OBDTM 10 μm ODS reversed-phase column (2.1 cm × 25 cm) using a gradient system. The crude peptide was purified at a flow rate of 10.0 mL/min using a gradient of 0–100% acetonitrile (0.1% TFA) and water (0.1% TFA) over 60 min and was then lyophilized to yield the pure cyclic peptide c[WH]_5_: MALDI-TOF) (*m*/*z*) for C_85_H_85_N_25_O_10_: calcd. 1615.6911; found 1615.3584 [M]^+^.

### 3.2. Cell Culture

Human leukemia CCRF-CEM (ATCC # CCL-119) and ovarian carcinoma SK-OV-3 (ATCC # HTB-77) cell lines were purchased from the American Type Culture Collection. Cells were grown on 75 cm^2^ cell culture flasks. The Roswell Park Memorial Institute (RPMI)-1640 medium was used for leukemia cells and McCoy’s 5A medium was used for SK-OV-3 cells. The medium was supplemented with 10% fetal bovine serum (FBS), 1% penicillin−streptomycin solution (10,000 units of penicillin, and 10 mg of streptomycin in 0.9% NaCl) in a humidified atmosphere of 5% CO_2_ at 37 °C. All bioassays were performed in triplicate. 

### 3.3. Cell Viability Assays Using MTS

The cytotoxicity assay was performed via an MTS proliferation assay in CCRF-CEM and SK-OV-3 cells. CCRF-CEM (40,000 cells) and SK-OV-3 (5000 cells) were incubated with 100 µL of complete media. The cells were seeded overnight in 96 well plates. The linear and cyclic peptides at concentrations (10, 50, and 100 µM) were added to the cells and incubated at 37 °C with 5% carbon dioxide for 3 h. Then, MTS reagent (20 µL) was added. The cells were incubated at 37 °C with 5% carbon dioxide atmosphere. The fluorescence intensity of the formazan product was measured at 490 nm using a Spectra Max M2 microplate spectrophotometer. The percentage of cell survival was calculated as [(OD value of cells treated with the test mixture of compounds) − (OD value of culture medium)]/[(OD value of control cells) − (OD value of culture medium)] × 100%.

### 3.4. Cellular Uptake Assay of Fluorescein-Labeled Molecular Cargo Molecules (F’-Phosphopeptide and F’-Emtricitabine) by Flow Cytometry

The cellular uptake assays were performed via FACS analysis method using SK-OV-3 cells in 6-well plates. The fluorescein-labeled phosphopeptide (F’-GpYEEI) or fluorescein-labeled emtricitabine (F’-FTC) was added to the well plates at a concentration of 5 µM, followed by adding the peptide to the well in each plate at a concentration of 50 µM. The cells were incubated at 37 °C with 5% carbon dioxide for 3 h. Incubated cells were digested with 0.25% trypsin in EDTA for 5 min to detach from the surface followed by addition of 2 mL of complete media to deactivate the trypsin. The cells were centrifuged at 1000 rpm and washed with PBS two times. Finally, the cells were re-suspended in 400 µL of flow cytometry buffer and analyzed by flow cytometry. The data presented are based on the mean fluorescence signal for 10,000 cells collected. All assays were performed in triplicate. We used flow cytometry (FACSVerse flow cytometer, San Jose, CA, USA) using an FITC channel and CellQuest software to analyze the results. The data were calculated based on the mean fluorescence signal for 10,000 collected cells that were performed in triplicate.

### 3.5. Mechanistic Cellular Uptake Assay of Fluorescein-Labeled Phosphopeptide in the Presence of Endocytic Inhibitors by Flow Cytometry

The cellular uptake of F’-GpYEEI was tested in the presence of c[WH]_5_ and different internalization inhibitors, such as chlorpromazine (30 μM), 5-(*N*-ethyl-*N*-isopropyl)- amiloride (EIA, 50 μM), methyl-β-cyclodextrin (2.5 mM), and chloroquine (100 μM). CCRF-CEM cells (3 × 10^5^ cells/well) were pre-incubated with inhibitors for 30 min. Then, the treatment, containing a mixture of F’-GpYEEI and c[WH]_5_ (5 μM and 50 μM, respectively), was added into each well. The treatment was incubated for 3 h. A similar FACS procedure as before was followed to wash the cells and resuspend them for the analysis. 

### 3.6. Live Cell Microscopy Imaging

SK-OV-3 cells were grown with antibiotic-free DMEM 24 h prior to the experiment in a glass bottom petri-dish (60 mm × 15 mm) of 5000 cells per mL of media. The F’-GpYEEI was incubated with cyclic [WH]_5_ for 30 min at room temperature. Then, the cells were treated with a mixture of F’-GpYEEI (5 μM) and cyclic [WH]_5_ (50 μM) in Opti-MEM for 3 h at 37 °C. After 3 h, the medium containing the treatments was removed, and PBS was added to cells. The cells were washed with PBS for two times. Images were acquired with BZ-X710 fluorescence microscope (Keyence, Osaka, Japan) at 100× magnification for brightfield and FITC channels. Images were merged and processed using Image J software to visualize the fluorescence uptake by cells.

## 4. Conclusions

In conclusion, two novel PDDS, namely cyclic [WH]_5_ and linear (WH)_5_, were synthesized, characterized, and purified for cellular uptake studies. Both peptides exhibited no significant cytotoxicity in both SK-OV-3 and CCRF-CEM cells up to the concentration of 100 µM after 3 h. The linear and cyclic peptides significantly improved the cellular uptake of F’-GpYEEI and F’-FTC in SK-OV-3 cells after 3 h of incubation compared to the parent compounds alone. The cyclic peptide was shown to be a more efficient molecular transporter compared to the corresponding linear peptide. The enhancement of the cellular uptake of both F’-GpYEEI and F’-FTC in the presence of [WH]_5_ supports the potential utilization of this class of peptides as PDDS. This work may provide insight for further development of peptides containing tryptophan and histidine as PDDS.

## Supplementary Materials

The following are available online at http://www.mdpi.com/1420-3049/23/7/1536/s1, Figure S1: MALDI mass spectra of linear (WH)_5_ peptide, Figure S2: MALDI mass spectra of cyclic [WH]_5_ peptide, Figure S3: Analytical HPLC chromatogram of linear (WH)_5_ peptide, Figure S4: Analytical HPLC chromatogram of cyclic [WH]_5_ peptide.
