# Supplementary material for: Efficient Intracellular Delivery of Cell-Impermeable Cargo Molecules by Peptides Containing Tryptophan and Histidine
Source: Molecules. 2018 Jun 26;23(7):1536. doi: 10.3390/molecules23071536 (PMC6100250; doi:10.3390/molecules23071536)
Supplement: Supplementary file 1 [file molecules-23-01536-s001.pdf]

Supplementary Information

Efficient Intracellular Delivery of Cell-impermeable Cargo Molecules by Peptides Containing Tryptophan and Histidine

Amir Nasrolahi Shirazi, Saghar Mozaffari, Rinzhin Tshering Sherpa, Rakesh Tiwari*, and Keykavous Parang*

Center for Targeted Drug Delivery, Department of Biomedical and Pharmaceutical Sciences, Chapman University School of Pharmacy, Harry and Diane Rinker Health Science Campus, Irvine, California 92618, United States

nasro100@mail.chapman.edu (A.N.S.); mozaf100@mail.chapman.edu (S.M.); sherp101@mail.chapman.edu (R.S.) tiwari@chapman.edu (R.T.); parang@chapman.edu (K.P.)

***** Correspondence: parang@chapman.edu; Tel.: +1-714-516-5489

Table of Contents:

	Page
**1. MALDI mass spectrum of linear and cyclic $(WH)_5$ peptides**	**3**
**2. Analytical HPLC chromatogram of linear and cyclic peptides, $(WH)_5$ and $[WH]_5$**	**5**

1. MALDI mass spectrum of linear and cyclic peptides

Figure S1. MALDI mass spectra of linear $(WH)_5$ peptide.

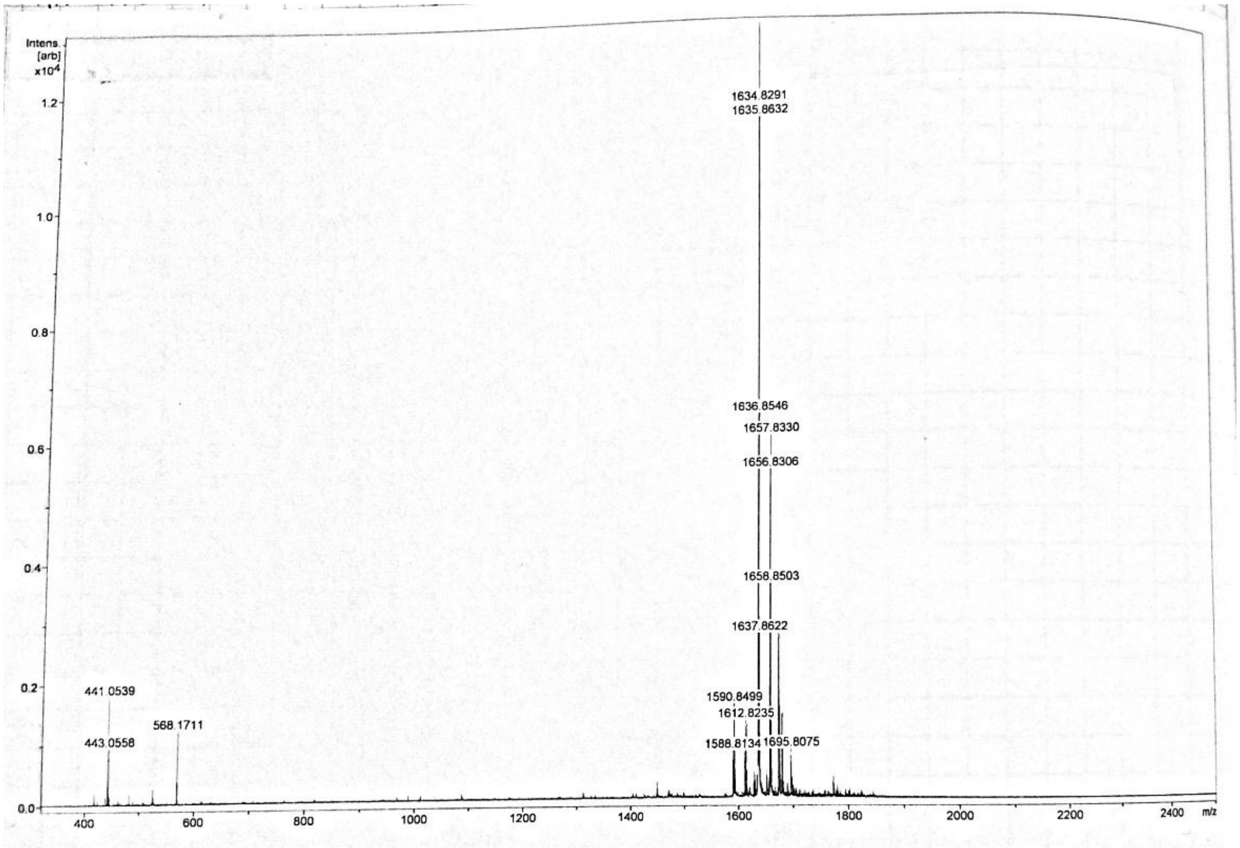

Figure S2. MALDI mass spectra of cyclic $[WH]_5$ peptide.

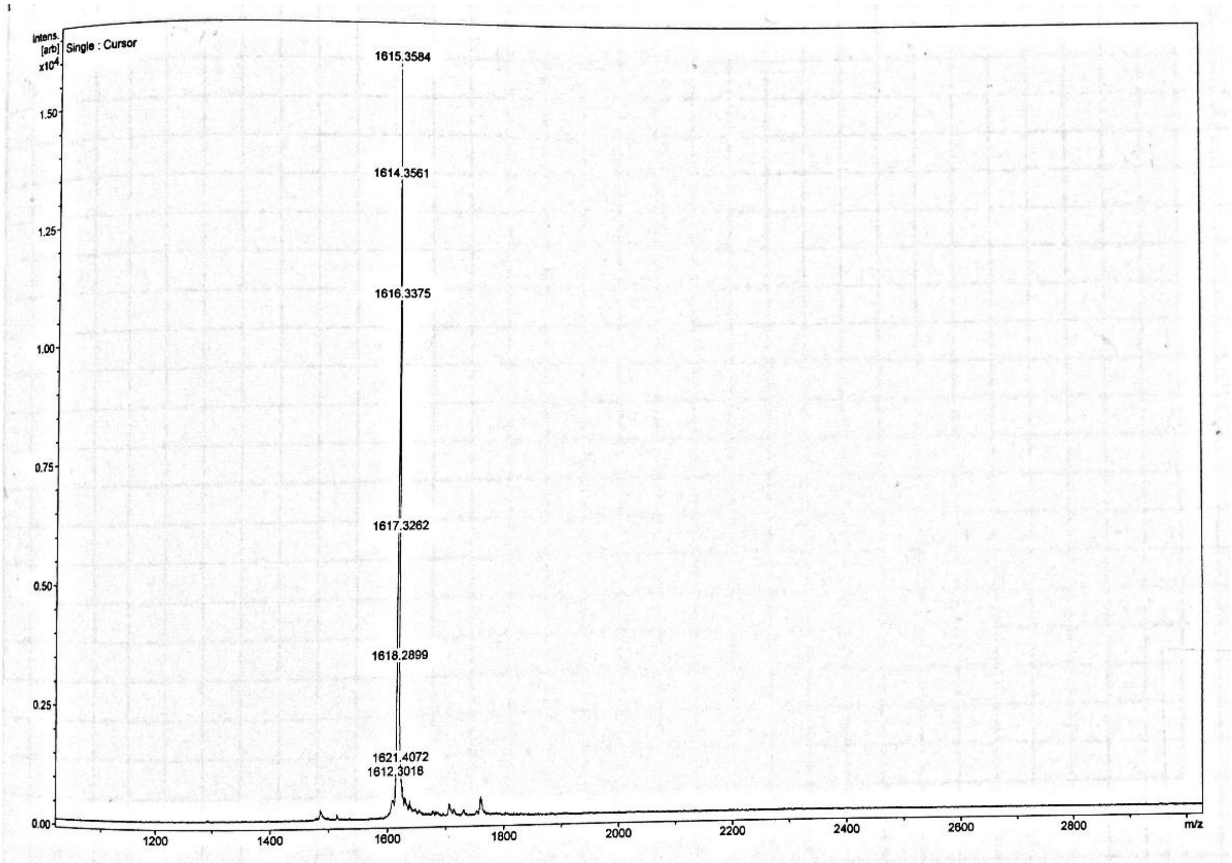

2. Analytical HPLC chromatogram of linear and cyclic peptides

Figure S3. Analytical HPLC chromatogram of linear $(WH)_5$ peptide.

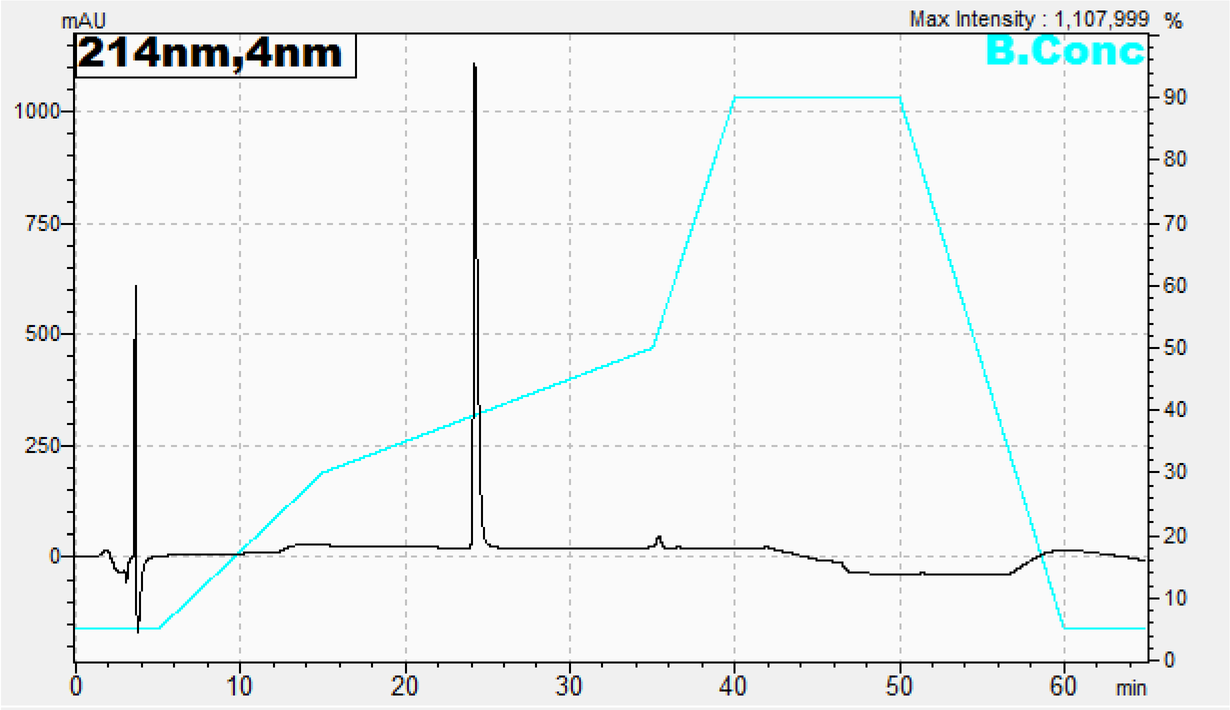

HPLC Method:

	Time	Module	Command	Value
1	5.00	Pumps	Solvent B Conc.	5
2	15.00	Pumps	Solvent B Conc.	30
3	35.00	Pumps	Solvent B Conc.	50
4	40.00	Pumps	Solvent B Conc.	90
5	50.00	Pumps	Solvent B Conc.	90
6	60.00	Pumps	Solvent B Conc.	5
7	65.00	Controller	Stop	

Figure S4. Analytical HPLC chromatogram of cyclic $[WH]_5$ peptide.

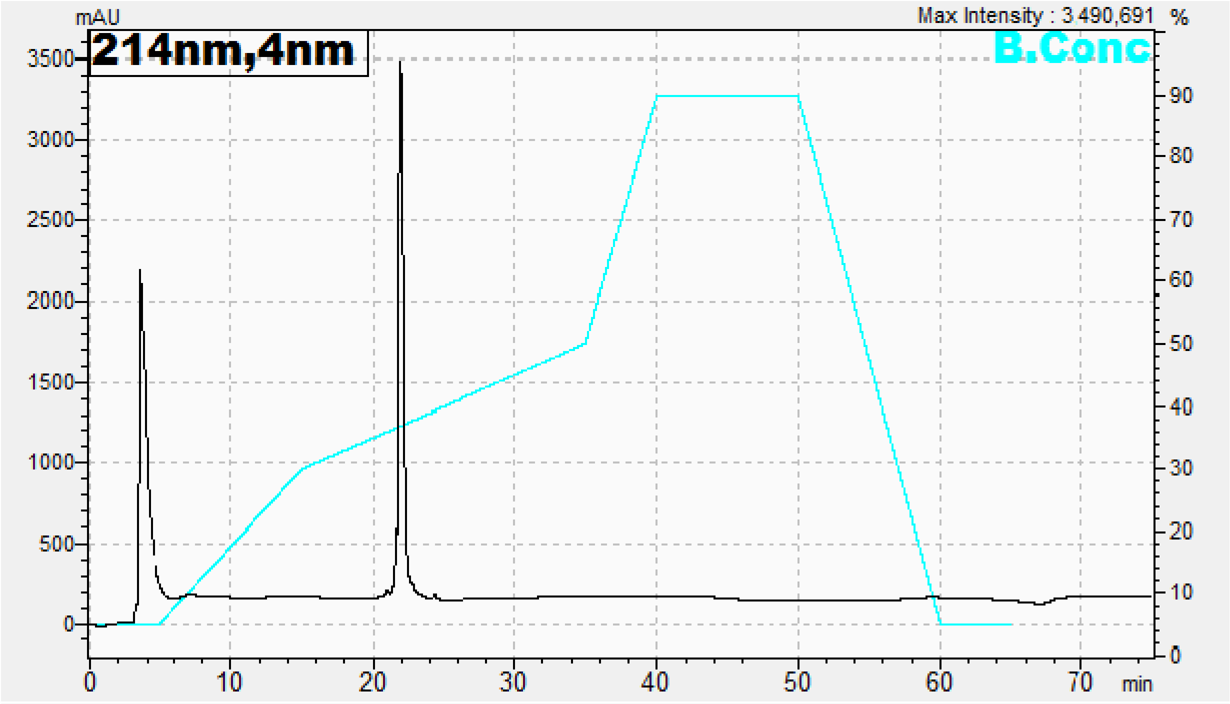

HPLC Method:

	Time	Module	Command	Value
1	5.00	Pumps	Solvent B Conc.	5
2	15.00	Pumps	Solvent B Conc.	30
3	35.00	Pumps	Solvent B Conc.	50
4	40.00	Pumps	Solvent B Conc.	90
5	50.00	Pumps	Solvent B Conc.	90
6	60.00	Pumps	Solvent B Conc.	5
7	65.00	Controller	Stop	
